# Parental Attachment Patterns in Mothers of Children with Anxiety Disorder

**DOI:** 10.3390/children7050046

**Published:** 2020-05-11

**Authors:** Şaban Karayağız, Timuçin Aktan, Lider Zeynep Karayağız

**Affiliations:** 1 Department of Psychology Kayseri/Turkey, Faculty of Science, Nuh Naci Yazgan University, 38170 Kocasinan, Turkey; taktan@nny.edu.tr; 2 Department of Psychology Tarsus/Turkey, Faculty of Science, Cag University, 33800 Tarsus/Mersin, Turkey; zohre-yildizi23@hotmail.com

**Keywords:** children with anxiety disorder, mothers, parental attachment, attachment styles

## Abstract

Anxiety disorder on of the most common illnesses in the context of psychiatry. Potential causes include genetic and environmental factors, as well as the parental attachment of the individuals. The purpose of this study is to examine the relationship between parental attachment style and anxiety disorders for a group of children and their parents. Study data were collected from the mothers (N = 40) of children with an anxiety disorder who visited a child psychiatry outpatient clinic at a city hospital and a private institution in Kayseri (Turkey) in 2018. For the control group, 40 mothers of children without any mental illness were also included in the study. The purposive sampling method was used in the selection of the participants for both groups (experimental and control). Sociodemographic data sheet and parental bonding instrument (PBI) were utilized as the data collection instruments. Then, data were analyzed based on the descriptive analysis methodology that included mean scores, standard deviation, *p*-value, t-experimental, two-way ANOVA and Pearson correlation experiments by using SPSS v.22. The findings revealed that the mothers of the participants with a college degree in the experimental group had fewer perceptions of protection (t = 2.38, *p* < 0.01), but more perception of care from their mothers than fathers (t =−2.28, *p* < 0.05). In addition, although the perceived care of parents was found lower than the participants in the control group, the participants in both groups evaluated their parents analogously for overprotection. Findings showed that the mothers in the experimental group predominantly described their parents as neglecting.

## 1. Introduction

Anxiety may be defined as the bodily response to a perception of danger. If it is exaggerated or overplayed in case of the perception of danger that influences functionality, it may lead to a disorder in the long term [1]. Contrary to some psychopathologies, anxiety refers to a feeling that has a part in both protection and adaptation of the individual throughout physical and mental developments. For example, the first day at school, sleeping alone or public speaking could make the individual tense or scared. However, if the feeling of anxiety persists and disturbs the quality of life and worsens family problems, the person may develop an anxiety disorder.

Even though anxiety disorder has been known since ancient times, comprehensive diagnostic and therapeutic studies started only in the first half of the 20th century [1]. It generally develops among the children with various types such as, “generalized anxiety disorder, panic disorder, social anxiety disorder and separation anxiety disorder.” This may trigger serious issues for the children—as well as their parents—and cause social problems in their mental developments that disturb the quality of life and raise in-house issues.

Anxiety disorders are common mental disorders in children and adolescents [2]. Its prevalence in the general pediatric population ranges between 5%–18% in the US [3,4]. The incidence of pre-adolescent children is lower (0.3%–12.9%) [5]. Specific phobias, separation anxiety disorder and generalized anxiety disorder are the most common forms of anxiety disorder [6]. Anxiety disorders are more common in girls [7]. In terms of age, the age of separation anxiety is lower than other anxiety disorders [8]. Some of the most common symptoms of anxiety in children include crying, unwillingness to go to school, fear, sadness, fear of separation, tension, restlessness, difficulty at focusing, difficulty at falling asleep, dry mouth and dizziness [9].

Studies of anxiety disorder in children have increased in recent years [10]. Some have been investigating its forms, upsetting factors and treatment methods [11,12,13,14,15,16]. We briefly examine significant findings of the previous studies regarding its patterns and stimulating factors.

Separation anxiety disorder (SAD) has been identified as the most common form of anxiety disorder observed during early childhood and influences approximately 4% of all children under 12 around the world [11]. In terms of treatment attempts, studies [12,13] have been conducted to test certain methods such as cognitive-behavioral therapy and drugs to reduce symptoms of the separation anxiety disorder among children.

One study [14] examined the prevalence of anxiety disorders and behavioral shyness among preschool children. Prevalence was found to be approximately 22.2%. Furthermore, in a recent study conducted in Turkey, the temperament levels of the mothers of the preschool children diagnosed with the anxiety disorder were investigated by observing the children and interviewing their mothers [15]. They observed the children and interviewed their mothers. It was discovered that the mothers had high levels of depressive, cyclothymic, irritable and anxious temperament. Furthermore, the existing mental problems of the mothers were the triggering elements of the anxiety disorder for the children. Similarly, other studies that investigated potential reasons behind anxiety disorder in children state that children’s mental health is disturbed by parents’ temperament and character traits [15].

Attachment is an important emotional concept for the individual, as it is based on relations of comfort, self-confidence and satisfaction [16,17]. It may be described as a cluster of strong emotional ties that people develop for individuals they care about. It is shaped by the nature of the relations with the caregiver. Its nature is specific to any kind of relationship. Patterns of attachment formed in early years are transferred to later periods of life through such models without undergoing a substantial change [18]. According to the attachment theory, attachment patterns are created based on the continuity and cognitive models of an individual’s cognitive abilities [19].

Bonding between the child and parent plays a crucial part in the healthy upbringing of a child and negative types of attachment influence an individual’s quality of life adversely in later years [19,20,21,22]. Children express their feelings of attachment for their parents through behavior, such as crying, calling, greeting, laughing.

Parental bonding instrument (PBI) is an important measurement scale regarding parental attachment perceptions. It measures attachment data from the retrospective perspective. It principally evaluates an individual’s perceived relationship with parents. It consists of two subscales: care and overprotection (or control). Subscales are measured for mother and father separately. In addition, four typical types of bonding features may be obtained with PBI to determine te parenting styles, such as high care and low protection—accepted as optimal parenting [22].

Parental attachment is defined as bonding between parent and child [22]. Attachment theory is based on the idea that there are individual differences in the way infants are emotionally attached to their caregivers, and this has impacts on their cognitive and emotional development [18]. Possible connections between attachment patterns of the caregiver and the anxiety disorder of a child may help us better understand this disorder and prevent possible psychopathologies that may develop in children in the future through changes in parental behavior.

Table 1 shows how children feel and perceived attachment in terms of four particular attachment styles and types of anxiety.

The securely attached child shows positive emotions to the family. It can be separated from parents without stress, seeks parents’ comfort when scared and positively responds to parents’ return. Children with an anxious attachment may be cautious around strangers and get very anxious as their parents leave. The avoidant child may neglect the family, does not want much care from their parents and does not care about strangers. Disorganized attachments display a mixture of indifferent and obsessive behaviors. They may be confused or worried. There is no clear-cut attachment behavior.

In light of the review of the literature, the following hypothesis were proposed: (1) A statistically significant difference exists between the patients’ mothers’ parental care in the experimental and the control group; (2) A statistically significant difference exists between the perceived care and protection scores of the mothers in the experimental group; (3) A statistically significant difference exists between the perceived care and protection scores of the mothers in the control group.

In relevant studies, the relationship between anxiety disorder and mother’s temperament [23], adolescent clinical characteristics [24,25], familial traits [26] and the incidence of mental disorder were investigated in Turkey. As no particular study previously focused on the relationship between the anxiety disorder of the children and the attachment patterns of their mothers to their parents, this study could be considered as the study of its kind in any country.

## 2. Materials and Methods

### 2.1. Ethics Approval

All subjects gave their informed consent for inclusion before they participated in the study. The study was conducted by the Declaration of Helsinki, and the protocol was approved by the Çağ University Institute of Social Science Research Ethics Committee Ref Number: 23867972/2015 (date 21/12/2018).

### 2.2. Setting

The sample of the study consisted of the mothers of children who were admitted to a city hospital and a private clinic in the city of Kayseri, Turkey, and diagnosed with an anxiety disorder by specialist child psychiatrists.

### 2.3. Participants

The criteria to be included in the study were being a caregiver and parent of a child diagnosed with an anxiety disorder. The participants were 80 mothers (40 in the experimental group and 40 in the control group). Table 2 summarizes their sociodemographic characteristics. A purposive sampling method was utilized to select suitable patients. Two requirements included being at the age of between 20 and 65 years old, and that the parent had a child diagnosed with an anxiety disorder.

### 2.4. Data Collection

A sociodemographic form was used to collect information about the participants involving education, occupation, marital status, number of children and income. The majority (77.5%) of the mothers in the study were middle-aged and their level of education was mostly (60%) high school or lower. The majority of them (88.88%) were married for 11–30 years with at least 2 children.

A second data collection tool, the parental bonding instrument (PBI), was utilized to gather data about parental attachment perceptions of the participating mothers. PBI was created by Parker et al. and translated into Turkish in 1979 [24,25,26,27]. It evaluates an individual’s perceived relationship with parents. The scale consists of 25 items and is administered separately for each parent. Each item is scored between 0–3 on a Likert scale. The maximum total score is 36. In the scale, 12 items (0–36 points) measure the dimension of care and 13 (0–39 points) measure the dimension of control/overprotection. Crossing the two dimensions, four types of parenting can be identified. Optimal parenting involves approaches with low protection and high care in raising children. Neglectful parenting corresponds to low protection and low care [22]. The parenting style with high protection and high care is described as affectionate constraint, while low care and high protection constitute the affectionless control. The cutoff points for these values on the relevant scale were 27 points for care and 13.5 points for protection in mother; and 24 points for care and 12.5 points for protection in father subsamples.

### 2.5. Data Analysis

Data were analyzed with the quantitative research analysis methods involving correlations and pairwise comparisons. Statistical analysis was performed with the SPSS v.20 software. When frequency distribution was done for the responses, a normal (Gaussian) distribution was formed. Therefore, parametric statistic experiments were conducted. Means and standard deviations, correlations, *p*-values (t-experimental) and F values were presented. Bounding types (care/protection-experimental; control) in the experimental and control groups were compared using a two-way ANOVA experimental.

## 3. Results

The sociodemographic information of the participants was shown in Table 3. Pearson correlation coefficient was used to calculate the correlation between the scores obtained from the PBI subscales in experimental and control groups as well as the education status, age, number of children and occupation of the parents.

Participants were divided into two groups according to their education level: high school or lower and university or higher degree (Table 3). A total of 35 (77.5%) were at the age between 36 and 64. Both the control group and experimental group shared similar distributions. In terms of their education status, 24 (60%) mothers were middle school or high school graduates in both groups.

It was also found that the majority (N = 21, 52.5%) of them in the experimental group were homemakers and the majority (N-25, 62.5%) in the control group were employed. However, the difference was not statistically significant (chi-squared = 1.82, *p* > 0.05). When compared in terms of the number of children, the chi-squared experimental showed that the number of mothers with 1 child in the experimental group was smaller than that of the control group and the number of mothers with 2 or 3 children was similar (chi-squared = 9.27, *p* < 0.01).

Table 4 shows the differences between education levels. Mean scores of the care and protection for the parents of the experimental group (19.92, 19.71) were much lower than that of the control group (28.51, 20.37) for the participants with a secondary or high school diploma. The parents of the mothers in both experimental and control groups with a college degree also showed similar patterns regarding their maternity care and protection. In addition, similar figures were observed for their fathers.

Particularly, PBI subscale scores calculated separately for experimental and control groups. It was found that participants with university or higher education perceived less protection from their mothers (t = 2.38, *p* < 0.01) and more care from their fathers (t (38) = −2.28, *p* < 0.05). Furthermore, the participants in the control group perceived more protection from their fathers than their mothers (t = −1.51, *p* < 0.05).

Table 5 shows the differences between employed and homemaker participants on PBI subscale scores calculated separately for experimental and control groups. The averages of the mother care and protection (18.86, 18.12) were found slightly higher for the homemaker group in the experimental group. However, the experimental group scored lower than the control group in terms of occupation forms.

The results also show that there was no significant relation between PBI subscales and occupation in the experimental and control groups (*p* > 0.05).

Table 6 shows the correlation coefficients between perceived parental care and perceptions for the participants. Even though the correlations were not significant, mother’s care was negatively correlated with mother overprotection (−0.13) and father overprotection (−0.12) in experimental group, while these correlations were positive in the control group.

The correlations of Mother care - father care and mother overprotection-father overprotection were significant in both the test group (Table 6). The values calculated were for the experimental group, *r_care_* = 0.45 (*p* < 0.001), *r*_overprotection_ = 0.52 (*p* < 0.001) and for the control group, *r_care_* = 0.42 (*p* < 0.001), *r*_overprotection_ = 0.56 (*p* < 0.001).

To investigate whether the participants in the experimental and control groups differed in terms of perceived care and overprotection from their parents a 2 (Group: experimental–control) X 2 (Parenting dimensions: care–overprotection) mixed ANOVA with a within-subject factor of parenting dimensions was conducted separately for PBI regarding mother and father. Parenting dimensions (styles) refers to PBI subscales measuring parents’ perceived care and overprotection of mother/father.

Table 7 illustrates the main factors for parental dimensions. As seen in Table 7, main effects of group and parental dimensions was significant for ratings regarding to both mother and father. More importantly, the group by parental dimension interaction was also significant for both ratings.

The main effect of group indicated that control group had higher ratings on both parental dimensions for mother (mean = 23.97) and for father (mean = 21.99) than control group (means = 18.63 and 18.49, respectively; Table 8). The main effect of parental dimensions showed that participants’ ratings of care for their mother (mean = 23.63) and father (mean = 22.41) was higher than their ratings of overprotection (means = 18.95 and 18.06, respectively).

As seen in the table above, pairwise comparisons were conducted to probe the interaction of group by parental dimensions (Table 8). An examination of the differences between care and protection perceived from mother showed that in the experimental group, the perceived care and overprotection did not differ, but in the control group, the difference between care (mean = 28.05) and overprotection (mean = 19.9) was significant (t (39) = 5.60). Pairwise comparisons revealed a similar pattern for ratings toward father, showing that the control group (mean = 25.33) scored higher than the experimental group (mean = 19.49) on perceived care (t (76) = 3.36, *p* < 0.001), but the groups did not differ on overprotection (mean = 18.64; mean = 17.49; t (78) = 0.70, *p* > 0.05, respectively). Finally, when the differences between the perceived care and protection from the fathers were examined, it was found that the care and overprotection in the experimental group did not differ, but that the participants in the control group perceived more care (mean = 25.33) than overprotection (mean = 18.64) from their fathers (t (38) = 4.01, *p* < 0.001).

A study of the distribution of parenting styles showed that those who evaluated their mothers as “optimal parent” (42.86% and 57.14%) and “loveless restriction” (44.19% and 55.81%) were similar in experimental and control groups (Table 9). However, it was seen that most those who qualified their mother as having “loving restriction” parenting style were in the control group (85.71%), whereas all those who reported that their mother was a negligent parent (n = 9, 100%) were in the experimental group. Finally, it was found that most of the participants in the experimental group perceived “loveless restriction” and “negligent parenting” styles (n = 33, 82.5%) in their mothers, whereas this rate was relatively low in the control group (n = 19, 47.5%).

When the same comparisons were made for the perceived parenting style from the father, it was found that “optimal parent” (45.45% and 54.55%) and “loveless restriction” (52.53% and 47.37%) styles were observed with similar frequency in the experimental and control groups. On the other hand, the “loving restriction” perceived from the father was observed more frequently in the control group (61.90%) and “negligent parenting” was more common in the experimental group (75.00%). Finally, it was found that most (66.67%) of the participants in the experimental group perceived “loveless restriction” and “negligent parenting” styles from their fathers, while this rate was lower in the control group (51.28%).

## 4. Discussion

The first aim of this study was to compare the attachment patterns between the mothers of children diagnosed with an anxiety disorder against the mothers of children with no mental illness. When the results were evaluated in general, the perceived parental care of the participants for the experimental (experimental) group was lower than that in the control group. However, both group members considered their parents alike for overprotection similar to the previous research findings [28].

In terms of parenting styles, it was observed that perceived parental styles were similar. Nevertheless, the number of “negligent parent” was higher and the number of the “loving restriction” was lower in the experimental group. Regarding negligent and restrictive loving parents, the attachment theory specifies that inconsistency and negligence from the caregivers likely to cause anxiety for the relationship with their parents [28].

A comparison of both groups also revealed that “loveless restriction” and “negligent parenting” styles were observed more frequently in the experimental group and less so in the control group. As previously reported the parents of mothers of children with anxiety disorders may be more negligent in terms of attachment patterns [29]. The attachment theory (Bowlby and Ainsworth) signifies loving parenting as the protection from an anxiety disorder at an early age [29]. Therefore, it is significant for the caregivers to properly treat (show love) the youngsters to protect them.

The fact that the “loving restriction” or restriction love style was observed more frequently in the control group. It may suggest that the high-care and low-protection style (optimal parenting) could be accepted for the Western societies in which individualist values are more prevalent [22]. However, it may not be considered as optimal parenting in collectivist cultures such as Turkish society. Instead, the “loving restriction” whereby families may indicate a high level of care and high protection may rather be perceived as the ideal parenting style in this cultural environment. In a similar study, it was reported that Turkish parents were more protective of their children than parents of different cultures [30].

These results show that parenting styles, as shown in the aforementioned similar studies, are related to anxiety and similar psychopathologies developing in children [21,22,23,24,25,26,27,28,29,30,31,32,33,34,35,36]. For example, early intervention in children with anxiety has been emphasized in the literature and it may reduce anxiety disorder [36]. Parental attachment theory similarly supports that early interventions pose a great deal of building an identity without any anxiety issues [30].

In contrast to similar research findings [29], we found that care and overprotection were not statistically significant for neither the mothers nor the fathers. This implies that these parenting characters may be independent of each other. In future studies with adolescents, this point may be further assessed to reveal any correlation between perceived care and protection for the parents. In conclusion, the overprotective parenting style engenders anxious and dysfunctional attitudes about dependency on gender-specific manners for the child toward caregivers. This study seemed not to support such a result of the theoretical perspectives of the previous research findings [37].

Four hypotheses were suggested at the beginning of the study concerning the potential relationships among the variables and two of them were supported by the study findings. For example, significant differences were found between the parent attachment patterns for the experimental and control groups. Additionally, a statistically significant difference existed between the mothers’ perceived care from their parents and protective values. However, the hypothesis about attachment subtypes was not supported by the findings as no significant difference existed between the care and protection values of the mothers between experimental and control groups.

Finally, this study is limited for the evaluated parents and the correlation appeared as a result of the retrospective evaluation. Additionally, this study was conducted with a quantitative research approach and under the rules of descriptive analysis so the findings somewhat may be extended to its setting and a similar group of parents. For further studies in the future, data could be collected not only from the mothers, but also from the fathers, because it could provide richer and deeper information.

## Figures and Tables

**Table 1:** Matrix of attachment styles and types of anxiety [21].

	Safe		Worrying	Abstaining	Disorganized
Separation anxiety	Worries when the mother leaves		Intensive anxiety when the mother leaves	No worry when the mother leaves	Abstaining, resistance and mixed responses
Stranger anxiety	Abstains, friendly to mother		Fears strangers, avoid them	Not affected by strangers	Wants to connect, but erects walls
Behavior when united	Happy		Approaches reject contact	Does not care	Does not care
Other	Mother is a safe place	Cries more and loses interest in the environment	Equal distance to mother and stranger	Frequent distress and anger	

**Table 2:** Demographic characteristics of the mothers (N = 80) in the study.

Demographic Characteristics	Categories	Control Group	Experimental Group	Total
		Frequency (%)	Frequency (%)	Frequency (%)
**Age**	Young adult (20–35)	5 (12.5)	5 (12.5)	10 (12.5)
	Middle-aged (36–64)	35 (77.5)	35 (77.5)	70 (77.5)
**Education**	Secondary or high school	18 (40.0)	24 (60.0)	42 (53.8)
	University	22 (55.0)	16 (45.0)	38 (46.2)
**Occupation**	Homemaker	15 (41.7)	21 (58.3)	36 (45.0)
	Employed	25 (56.8)	19 (43.2)	44 (55.0)
**Marital Status**	Married	35 (49.3)	36 (50.7)	71 (88.8)
	Divorced	5 (55.6)	4 (44.4)	9 (10.0)
**Number of Children**	1	14 (82.4)	4 (17.6)	18 (21.8)
	2	16 (39.0)	25 (61.0)	41 (52.6)
	3	8 (40.0)	12 (60.0)	20 (25.6)
**Marriage Duration (Years)**	0–10	7 (63.6)	5 (36.4)	12 (16.9)
	11–20	12 (38.7)	19 (61.3)	31 (43.7)
	21–30	16 (59.3)	11 (40.7)	27 (38.0)
	30+	0	1 (100)	1 (1.4)

**Table 3:** Sociodemographic characteristics of the experimental and control groups.

Study (Experimental) Group				Control Group				Chi-Square
S. Characteristics	N	%	Mean	S. Characteristics	N	%	Mean	
**Age**				**Age**				0.00
20–35	5	12.5		20–35	5	12.5		
36–64	35	77.5		36–64	35	77.5		
			40.9				39.9	
**Education Level**				**Education Level**				1.80
Secondary/High	24	60		Secondary/High	18	45		
School				School				
College	16	40		College	22	55		
**Occupation**				**Occupation**				1.82
Homemaker	21	52.5		Homemaker	15	37.5		
Employed	19	47.5		Employed	25	62.5		
**# of Children**				**# of Children**				9.27 **
1	3	7.5		1	14	35		
2	25	62.5		2	16	40		
3	12	30		3	10	25		
**Years in Marriage**				**Years in Marriage**				2.47
1–9	5	14		1–9	7	14		
10–19	19	53		10–19	12	33		
20+	12	33	16.3	20+	16	43	19.25	

** *p* < 0.01; #: This define how many children participant have.

**Table 4:** Differences between education levels and parent attachment patterns of participants (t-experimental for independent groups).

	Experimental Group					Control Group				
Education	Secondary or High School		University or Higher		T	Secondary or High School		University or Higher		T
	Mean	SD	Mean	SD		Mean	SD	Mean	SD	
**Mother**										
Care	19.92	5.68	18.12	5.71	0.98	28.51	5.87	27.68	6.01	0.44
Protection	19.71	5.38	15.56	5.42	2.38 **	20.37	6.02	19.53	6.12	0.44
**Father**										
Care	17.92	5.21	21.85	5.41	−2.28 **	26.42	5.78	24.43	5.82	1.08
Protection	18.47	5.23	16.01	5.38	1.43	17.09	5.73	19.91	6.03	−1.51 *

* *p* < 0.05, ** *p*< 0.01. SD: Standard Deviation. T: Total.

**Table 5:** Differences between occupations and parent attachment patterns of participants (t-experimental for independent groups).

	Experimental					Control				
Occupation	Homemaker		Employed		T	Homemaker		Employed		T
	Mean	SD	Mean	SD		Mean	SD	Mean	SD	
**Mother**										
Care	19.86	5.23	18.47	5.29	0.83	28.45	5.42	27.81	5.36	0.36
Protection	18.12	5.19	17.98	5.21	0.09	20.19	5.31	19.72	5.16	0.27
**Father**										
Care	19.51	5.27	19.46	5.21	0.03	24.97	5.14	25.55	5.08	−0.35
Protection	17.12	5.13	17.91	5.16	−0.48	19.61	5.27	18.06	5.14	0.91

**Table 6:** Correlations between perceived parental care and perception.

	Mother		Father	
	Care	Overprotection	Care	Overprotection
**Mother**				
Care		−0.13	0.45 **	−0.12
**Overprotection**	0.10		−0.05	0.52 **
**Father**				
Care	0.42 **	0.29		−0.23
**Overprotection**	0.25	0.56 **	−0.05	

** *p* < 0.01. The top gray section in the table shows the correlations obtained from the experimental group.

**Table 7:** Examining the main factor of parental dimension.

		Sum of Squares	DF	Mean Squares	F	*P*
**Mother**	**Group**	864.90	1	864.90	17.09	0.000
	**Parental Dimensions**	1144.90	1	1144.90	23.53	0.000
	**Group X Parental Dimension**	490.00	1	490.00	9.68	0.003
	**Error**	3947.10	78	50.60		
**Father**	**Group**	736.67	1	736.67	11.42	0.001
	**Parental Dimensions**	477.75	1	477.75	3.33	0.072
	**Group X Parental Dimension**	214.67	1	214.67	9.95	0.002
	**Error**	4901.15	76	64.49		

DF: Degrees of Freedom.

**Table 8:** Differentiation between experimental and control groups in terms of the perceived parental care and overprotection (ANOVA findings).

	Experimental		Control		Total		Independent Groups T
	Mean	SD	Mean	SD	Mean	SD	
**Mother**							
Care	19.20	7.11	28.05	7.47	23.63	7.29	5.43 ***
Overprotection	18.05	7.34	19.90	6.19	18.95	6.79	1.22
Total	18.63	4.93	23.97	4.93			
**Father**							
Care	19.49	8.13	25.33	7.21	22.41	7.78	3.36 ***
**Overprotection**	17.49	7.47	18.64	7.16	18.06	7.41	0.70
**Total**	18.49	4.96	21.99	4.96			

*** *p* < 0.001.

**Table 9:** Distribution of participants according to the parenting style of their parents.

	Control		Experimental		Total
	N	%	N	%	
**Mother**					
**Optimal parenting**	3	42.86	4	57.14	7
**Loving restriction**	18	85.71	3	14.29	21
**Loveless restriction**	19	44.19	24	55.81	43
**Negligent parenting**	0	0.00	9	100.00	9
**Father**					
**Optimal parenting**	6	54.55	5	45.45	11
**Loving restriction**	13	61.90	8	38.10	21
**Loveless restriction**	18	47.37	20	52.63	38
**Negligent parenting**	2	25.00	6	75.00	8
